# Low Cost and Lithography-free Stamp fabrication for Microcontact Printing

**DOI:** 10.1038/s41598-018-36521-x

**Published:** 2019-01-31

**Authors:** Akshada J. Khadpekar, Moin Khan, Abhishek Sose, Abhijit Majumder

**Affiliations:** Indian Institute of Technology Bombay, Mumbai, 400076 India

## Abstract

Microcontact printing (µCP) is a commonly used technique for patterning proteins of interest on substrates. The cells take the shape of these printed patterns. This technique is used to explore the effect of cellular morphology on their various functions such as survival, differentiation, migration, etc. An essential step for µCP is to fabricate a stamp from a silicon mould, prepared using lithography. Lithography is cost intensive and needs a high level of expertise to handle the instrumentation. Also, one stamp can be used to print patterns of one size and shape. Here, to overcome these limitations, we devised a low-cost fabrication technique using readily available objects such as injection needles and polystyrene beads. We patterned the C2C12, myoblasts cells on the shapes printed using lithography-free fabricated stamps. We further exploited the surface curvature of the stamp to vary the size of the print either by changing the applied load and/or the substrate stiffness. We showed that the print dimension could be predicted well by using JKR theory of contact mechanics. Moreover, some innovative improvisations enabled us to print complex shapes, which would be otherwise difficult with conventional lithography technique. We envisage that this low cost and easy to fabricate method will allow many research laboratories with limited resources to perform exciting research which is at present out of their reach.

## Introduction

Surface patterning by selective chemical functionalization is widely used in electronics^1^ and biomedical research^2^. Depending upon the final application and length scale of the pattern, various methods are employed such as patterning using membranes^3^ or poly-(dimethyl siloxane) (PDMS) stencils^4^, selective UV^5^ exposure, direct deposition using AFM tip^6^, surface wettability guided patterning^7^, microfluidics^8–10^ based patterning, and microcontact printing (µCP)^11–13^. Out of all these methods, µCP is probably the most widely used technique in the field of cell biology to understand the effect of cell shape and size on cell growth and survival^14^, differentiation of stem cells^15,16^, contractility of the cell^17^. Besides these, µCP is also employed in biomedical research such as DNA hybridization^18–20^ targeted differentiation into cells with specific properties for engraftment^21^, controlling the size of embryoid bodies^22^ and much more.

The key steps involved in microcontact printing (µCP) are (1) fabrication of stamps with pre-designed pattern, (2) inking the stamp with desired bio-molecules and (3) transferring the bio-molecues onto the desired substrate. Out of these three, the most challenging task is to fabricate the stamp with precise pattern i.e. the step 1 using photolithography^23^. Photolithography is the technique which makes use of light-sensitive resist layers to transfer the pattern drawn on the mask to silicon template. However, there are certain disadvantages of this technology. For example, (i) it is not applicable for nonpolar and curved surfaces, (ii) the etching of mask depends on diffraction of light, and iii) to develop a pattern on the mask, only photosensitive polymers can be used. Whiteside *et al*. developed a technique, soft lithography, which circumvents some of the disadvantages of photolithography^24^. The soft lithography technique is used to generate patterns and structures on non-planar surfaces and can be utilized for biological materials. In this technique, elastomers such as Poly(dimethylsiloxane) (PDMS) is used for moulding the silicone template for stamp preparation. Although there are several advantages of soft lithography over photolithography, it is still dependent on a photolithographic method for fabrication of the starting template. The requirement of the expertise to design the pattern and template fabrication makes it difficult for a researcher to use this technology. Also, the need for sophisticated instrumentation and clean room makes this technique less accessible to the labs where the limited funding is a serious concern. Besides these, lifting off the PDMS from the template may lead to distortion of the stamp and block the master plate, ultimately making it non-reusable. Moreover, if one wants to vary single parameter like size or shape of the print, it cannot be done using the same stamp, and fabrication of a new stamp is required.

To overcome these bottlenecks, the goal of this study was to demonstrate a method to fabricate low-cost stamps, independent of sophisticated instrumentation and expertise. This goal was accomplished by using easily available materials such as injection needles and polystyrene microbeads to fabricate the stamps. While micro-beads have been used to create porous micro-structures^25^ and 3D microwells^26^ earlier, to the best of our knowledge, this is the first work to use them for µCP. In addition, taking the advantage of the curvature of beads and cylindrical PDMS stamps, we exploited JKR contact mechanics^27^ to obtain prints of variable sizes using the same stamp. We changed the applied weight on the stamp and/ or the substrate stiffness on which the pattern is transferred in order to get different contact area, hence different print sizes. We also used the slender PDMS cylinders to create other complicated patterns which are challenging to obtain otherwise. Finally, we have shown that the cells remain viable on the pattern thus obtained and take shape of the patterns.

## Results

### Cell patterning using lithography-free fabricated stamps

**Figure 1:**
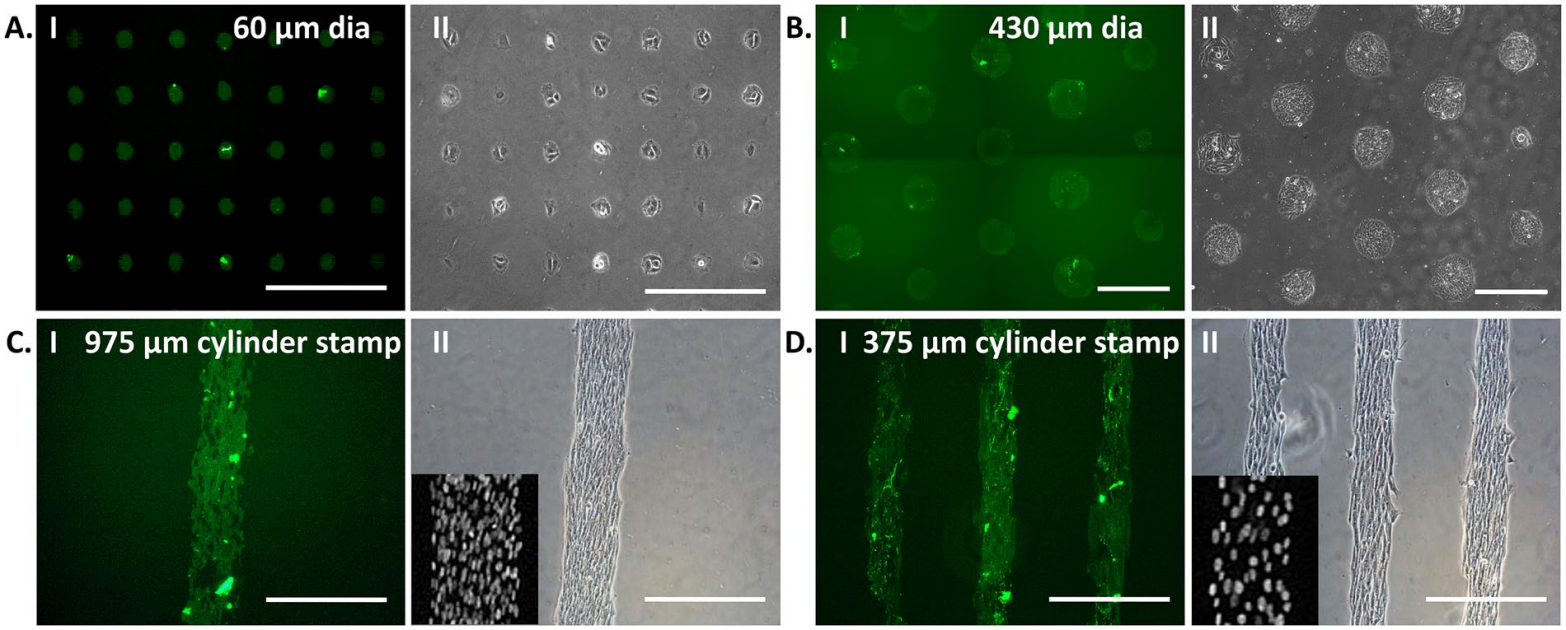
Patterning of C2C12 myoblasts on FITC-conjugated collagen-I islands: Protein was printed using a stamp of 60 µm diameter fabricated with soft lithography (**A**-I) and manually fabricated in the lab, i.e., 430 µm diameter polystyrene beads stamp (**B**-I), 975 µm (**C**-I) and 375 µm (**D**-I) diameter PDMS cylinders stamp. C2C12, mouse myoblast, were then cultured on the protein patterned substrates using 60 µm diameter PDMS stamp (**A**-I I), 430 µm diameter polystyrene beads stamp (**B**-I I), 975 µm (**C**-I I) and 375 µm (**D**-I I) diameter PDMS cylinders stamp. On cylindrical patterns, the cells were aligned longitudinally. The nucleus of aligned cells stained with Hoechst is shown in the inset images (**C**-I I and **D**-I I). [Scale bar = 400 µm].

Plastic petri-plates, printed with FITC-conjugated Col-I (Fig. 1A-I,B-I,C-I,D-I) as described in materials and methods section, was used for cell-patterning. As shown in Fig. 1A-II,B-II,C-II,D-II, C2C12 cells, a mouse myoblast cell line, were patterned on the protein islands. In Fig. 1A, C2C12 cells were restricted in the circular protein islands printed using 60 µm diameter stamp, made by conventional soft lithography. Figure 1B shows the result from lithography-free fabricated 430 µm diameter polystyrene bead stamp when printed on PDMS (1 MPa) substrate. The mean island size obtained here was 256 ± 27 µm. To note, print on plastic with stamps made from beads resulted only point contacts as both the beads and the substrates were rigid and non-deformable. We also used stamps made of PDMS cylinders with 975 µm and 375 µm diameter for patterning C2C12 on non-tissue culture plastic dishes and the pattern size was 181 ± 10 µm and 127 ± 8 µm, respectively (Fig. 1C-I,D-I). When we changed the geometry from a circle to a rectangle, cells got aligned along the length of the rectangular print (Fig. 1C,D).

We also prepared stamps using needles of other gauge sizes and printed protein, just to confirm the range of applicability of our process (see Supplementary Fig. S1). Further, to estimate the variation due to manual error from one fabrication cycle to the other, we prepared two stamps independently from same size needles and printed protein on plastic dishes. It was found that there was no significant difference between the print width, obtained from two individually fabricated stamps (see Supplementary Fig. S2).

Next, to compare the reliability of our process vis-a-vis conventional stamps, we compared size of the islands printed using stamp fabricated by lithography technique as control with the same using stamps made by our method, both in identical conditions (on PDMS substrate of 1 MPa stiffness, putting 20 g weight) (see Supplementary Fig. S3). It was found that the average deviation from the mean size was 3.1% for control and 10.7% for the stamp made using the bead.

Similarly, we compared the uniformity in protein deposition by these two methods. We found that the average deviation from the mean intensity was 11% for control and 24% for the stamp made using the beads (see Supplementary Fig. S3).

Finally, to confirm the cell viability on the printed islands, we captured the images of C2C12 cells on prints on plastic (375 μm cylinder stamp, 20 g weight) after 12 h and 24 h of cell seeding. As it can be seen in Supplementary Fig. S4, the cells proliferated over time, and partially covered the patterned area indicating good cell health. Further, we have also confirmed the cell viability by using calcein/PI staining. The live cells take up the calcein dye whereas PI stains the dead cells. As shown in Supplementary Fig. S4, all the cells on the pattern are viable as they all are positive for calcein but negative for PI staining.

### Printing pattern of different size by exploiting the curvature of the lithography-free fabricated stamps

**Figure 2:**
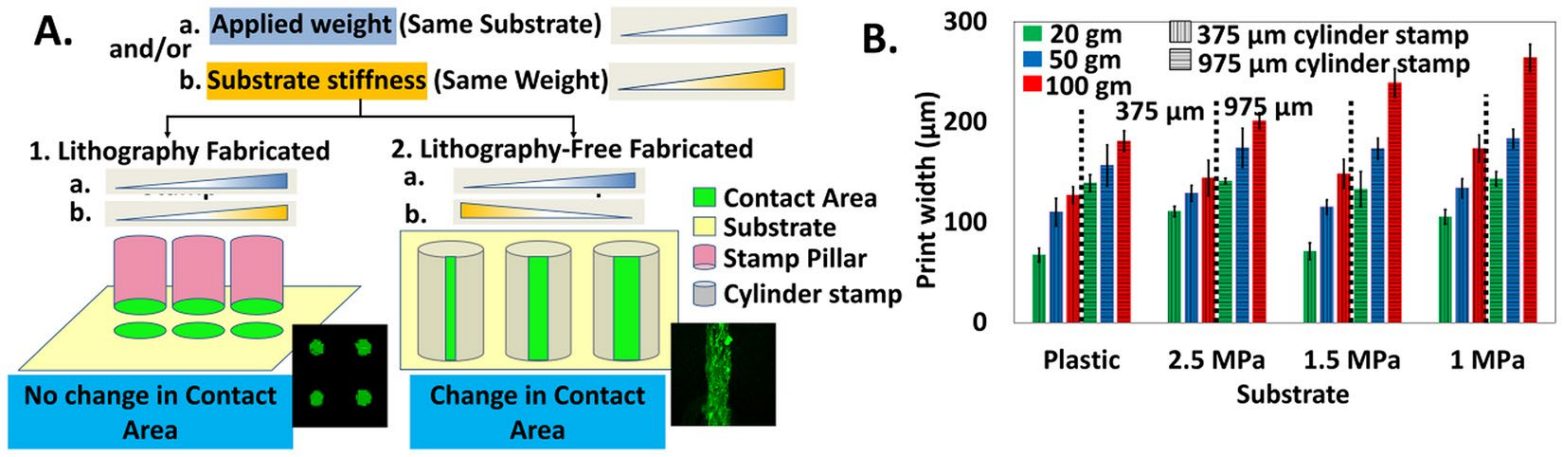
Variation in print width (contact area) depending on applied load and/or substrate stiffness. (**A**) Schematic representation of contact area obtained by varying applied weight (a) and/or substrate stiffness (b) on lithography fabricated stamps pillar with flat bottom (1) and lithography-free fabricated PDMS cylinder stamps with curved bottom (2). For (1), the contact area is independent of applied weight and varying substrate stiffness whereas for (2) the contact area increases with increasing applied weight on the same substrate and/or decreasing substrate stiffness using same weight. (**B**) Print width obtained using lithography-free fabricated 375 µm and 975 µm PDMS cylinder stamps of diameter on four substrates with varying stiffness (Plastic, 2.5 MPa, 1.5 MPa, 1 MPa and three different applied loads (20 g, 50 g, and 100 g). On each substrate, the size of the print increases with increasing the applied load. Also, for 100 g applied load, and 975 µm diameter PDMS cylinder stamps, the print size increases as the substrate become softer. (n ≥ 6 cylinders).

As shown in schematic in Fig. 2A, using a conventional stamp with flat printing surface, we can obtain the pattern of only one size which does not vary with either applied weight and/or substrate stiffness. However, between a curved (here cylindrical stamp) and the flat substrate, the print size is the function of applied weight and/or substrate stiffness if either or both contacting body is deformable. Using this logic, we applied weights of 20 g, 50 g and 100 g on PDMS cylinder stamps of 975 µm and 375 µm diameter for printing on substrates with four different rigidity ranging from stiff to soft. The substrates used were untreated plastic and PDMS of different stiffness.

We observe that for both 975 µm and 375 µm stamp, print width increases with increasing load for the substrate of all four stiffness (Fig. 2B). However, a variation of print width with substrate stiffness for constant applied weight is not that straightforward.

### To predict the print size using JKR theory of contact mechanics

**Figure 3:**
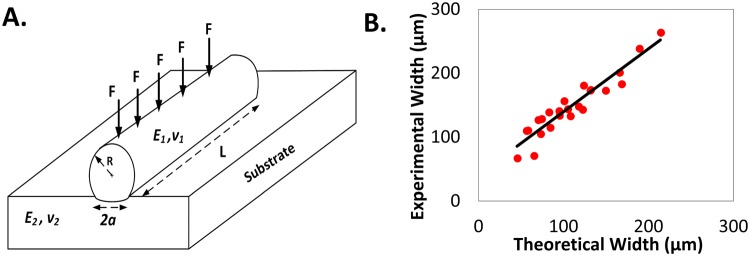
Comparison between theoretical and experimental values of print width. (**A**) The schematic of contact between PDMS cylinder and the flat substrate. (**B**) Comparison between the theoretical print width and the Experimental print width. There is a linear relationship between the two.

Further, we wanted to check if we can predict the print width beforehand enabling a researcher to design her stamp more efficiently. When two rigid objects are in contact, a ‘frictionless’ assumption is made at the contact surface, so that shear stresses cannot be sustained at the interface, accounting only for compressive forces^27^. For our system, shown in Fig. 3A, such an assumption falls apart due to the onset of significant adhesive forces. These adhesive forces play a major role in determining contact area between the two objects^27^. We used the formula,

$$P=\frac{(1-4{\rho }_{0}^{2})\pi {E}^{\ast }{a}^{2}}{4R}-\sqrt{2\pi {E}^{\ast }a\Delta \gamma -{(\frac{{\rho }_{0}\pi {E}^{\ast }{a}^{2}}{R})}^{2}}$$

where, *P* is the force per unit length, acting on one cylinder, *R* is the radius of cylinder, *Δγ* is the adhesion energy of two solids with surface tensions γ_1_ and γ_2_; *2a* is the width of stamping; E* is an effective modulus of the stamp-substrate system^28^, to calculate the values of expected print width for 24 conditions (2 stamps X 3 weights X 4 substrate stiffness).

To calculate *P*, the weight of the load placed above a stamp was divided by the number of PDMS cylinders present in a stamp (8 in case of the 975 µm stamp, and 9 in case of 375 µm stamp). This force was then divided by the length of the cylinder (1 cm) in contact with the substrate

Δγ was calculated using the relation:

$${\boldsymbol{\Delta }}{\boldsymbol{\gamma }}={{\boldsymbol{\gamma }}}_{{\bf{1}}}+{{\boldsymbol{\gamma }}}_{{\bf{2}}}-{{\boldsymbol{\gamma }}}_{{\bf{12}}}$$

where γ_12_ is the interfacial tension (γ_12_ is very small as compared to γ_1_ or γ_2_ for PDMS-PDMS and plastic-PDMS cases, therefore is neglected).

***ρ***_**0**_ is a function of Poisson’s ratio of the substrate and is calculated using the formula,

$${{\boldsymbol{\rho }}}_{{\bf{0}}}=\frac{{\bf{1}}}{{\bf{2}}{\boldsymbol{\pi }}}\,{\bf{l}}{\bf{n}}\,({\bf{3}}-{\bf{4}}{\nu }_{2})$$

where ν_2_ is Poisson’s ratio of the substrate.

E* is a function of elastic constants *E*_1_ and *E*_2_, as well as Poisson’s ratio *ν*_1_ and *ν*_2_ of the stamp and substrate, respectively. It is calculated using following formula,

$$\frac{{\bf{1}}}{{{\boldsymbol{E}}}^{\ast }}=\frac{{\bf{1}}-{{\boldsymbol{v}}}_{{\bf{1}}}^{{\bf{2}}}}{{{\boldsymbol{E}}}_{{\bf{1}}}}+\frac{{\bf{1}}-{{\boldsymbol{v}}}_{{\bf{2}}}^{{\bf{2}}}}{{{\boldsymbol{E}}}_{{\bf{2}}}}$$

We plotted the experimental width against the theoretical width and found a reasonable good linear fit with R^2^ = 0.78 and slope as 1.3 (Fig. 3B). A little higher width obtained from experiment might be due to wetting of the surface with the excess protein solution.

### Patterning complex shapes using stamps made of slender PDMS cylinders

**Figure 4:**
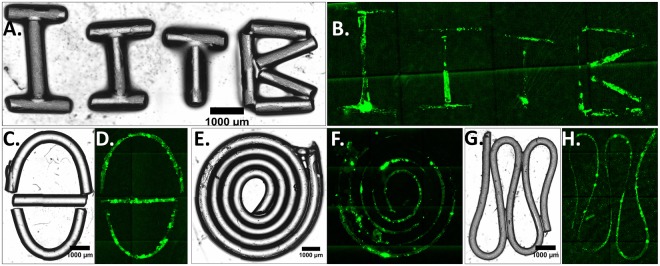
Patterning protein using unconventional stamps fabricated using PDMS cylinders: Bright-field images of letters (**A**), half circle (**C**), Spiral (**E**), and periodic wave (**G**) stamps fabricated using PDMS cylinder made using injection needles. The stamps were coated with FITC- conjugated Collagen-I protein and were printed on plastic. (**B,D,F,H**) are the protein print images obtained after µCP using (**A,C,E,G**) stamps, respectively. Since the size of the stamps is large, the images of stamps and print are stitched images using EVOS FL auto microscope.

After making prints of simple structures like circular islands and stripes, we proceeded further to make more complex patterns. Here, we used slender PDMS cylinders made using needles as a mould to fabricate the stamps with the sharp turns and curvature. As shown in Fig. 4, the PDMS cylinders were arranged as IITB letters (Fig. 4A), half-circles writing ‘Theta (θ)’ (Fig. 4C), Spiral with continuously changing radius of curvature (Fig. 4E) and a periodic wave (Fig. 4G) shape and used for patterning the protein on plastic (Fig. 4B,D,F,H).

## Discussion

In this study, we developed a low-cost, lithography-free stamp fabrication method using easily available objects such as injection needles and beads. To confirm the quality of printing, we patterned C2C12 myoblast cells on the printed substrates and showed that the cells were restricted only to the patterned regions. To note, although we have used the substrates of different rigidity, cell behavior is expected to be insensitive to any variation in substrate stiffness at that range. As a result, any observed difference in cell behavior can safely be attributed to the difference in pattern size and not to the substrate modulus. We have also shown that the print width can be changed by varying weight on the stamp and the width can be predicted by JKR contact mechanics. This essentially enables us to predetermine the needle size and weight for the desired print width. As our result shows, by using only two sizes of needles, one can obtain any print width ranging from 70 to 270 µm.

Our method is different from all existing methods of µCP in three aspects: 1. cost, 2. ease of fabrication and 3. versatility of print size using the same stamp. To compare, we estimated the cost of a silicon wafer template used in conventional lithography and needles and beads used in our case. A silicon wafer with desired pattern fabricated, costs around $22 in IIT Bombay microfabrication facility at subsidized rate. It should be noted that the above-mentioned costs are only the consumable cost. However, the main bottleneck for conventional µCP is the huge infrastructural investment which often runs into tens of thousands of USD annually for an academic university^29^. Cost of a commercially available stamp is also very high (~$200–$500) (https://researchmicrostamps.com). In comparison, the material that we used in our method that is needles and beads, cost only ~$0.125 (www.smartmedicalbuyer.com) and ~$5 per stamp, respectively. The other methods of cell patterning such as using AFM tip, stencils or microfluidic channel also requires high end facilities such as clean room, ion beam, mask aligner, AFM, deposition system, etching set-up, high temperature pyrolysis oven, etc.^3,4,6,8^. Our method does not demand any such sophisticated facility. Moreover, micro fabrication processes generally involve intermediate materials which are toxic and carcinogenic. Our process involves no such harmful chemicals.

The second novelty factor of this work is the ease of fabrication. Making stamps with complicated curvatures is a daunting task using conventional lithography because of multiple technical challenges. Even with the most sophisticated infrastructure and high level of technical skills, it may not be possible to fabricate a complicated yet structurally robust stamp. In this work, we overcome that challenge by arranging slender PDMS cylinders. Patterns printed with such structures (Fig. 4B,D,F,H) with multiple curves can be very useful to study cellular migration or to explore the effect of the radius of curvature of a growing tissue on cellular differentiation.

Lastly, in conventional method, to get the print of varying size we need different stamps. Here, we showed that by using the stamp with a curved bottom, we can obtain the broad range of print size by varying the applied weight and/or by varying the substrate stiffness. We have also done the theoretical calculations using JKR theory of contact mechanics and showed that we can predict the variation of print size with substrate stiffness and/or applied weight.

One major limitation of our technique described in this paper is that we can only create circular or stripe-shaped protein islands. It is not possible to print any other shape such as triangle or star using this technique in its present form. We also do not have control over the packing of the micro-beads which is governed by surface energy minimization. However, we do not consider that as a major limitation because cellular behavior is expected to depend on size and shape of the individual printed island.

To conclude, here we present a novel method of µCP, strength of which lies in its comparatively easy and inexpensive stamp fabrication process that does not depend on sophisticated high-end infrastructure. This method can be used as a first pass checking of the effect of cell shape and size on cellular behavior. Also, patterns of multiple sizes are possible to print using the same stamp by exploiting its curved surface and JKR contact mechanics. As a result, the laboratories with low funding or the universities without such high-tech facility, particularly from the underdeveloped or developing countries, may now find it possible to explore many questions related to cell mechanics.

## Materials and Methods

### Lithography-free stamp fabrication method

**Figure 5:**
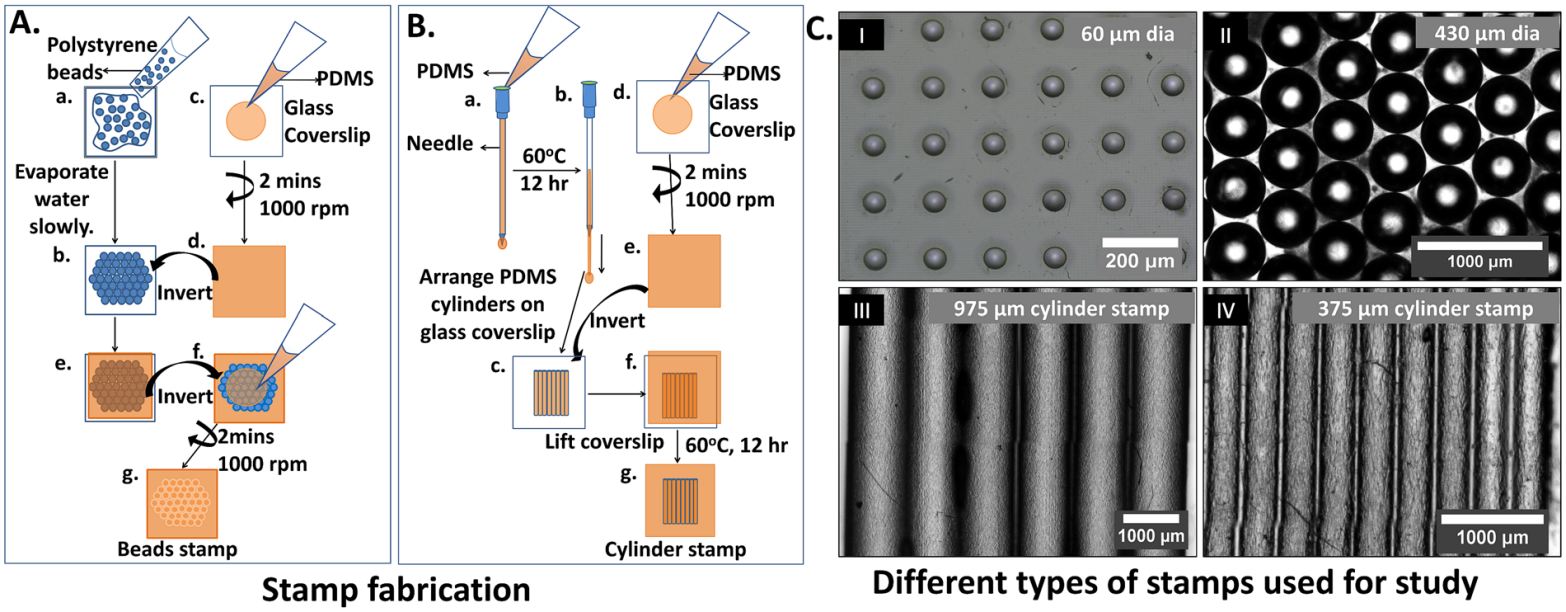
Lithography-free method for stamp fabrication: Schematic representation of the fabrication of stamps prepared using polystyrene beads (**A**) and PDMS cylinders (**B**). Representative images of stamps (**C**): (I) 60 µm diameter PDMS stamp fabricated using soft lithography, (II) 430 µm diameter polystyrene beads stamp, (III) 975 µm and (IV) 375 µm diameter PDMS cylinders stamp fabricated in the lab using the lithography-free method.

To prepare low-cost stamps, in this paper we have used two types of objects; one, polystyrene beads to get circular prints and two, syringe needles to get stripe-shaped prints. To describe the method in brief, for polystyrene bead stamps (Fig. 5A), a 10 µl drop of polystyrene beads suspension (Sigma, Cat. no. 75911) (430 µm diameter) was added on the glass coverslip and was allowed to dry by placing it on a hot plate at 37 °C for 2–3 h. The beads self-assemble into a hexagonal close packing when gently tapped. Packing efficiency improves further during drying due to minimization of surface free energy. A video of the process is shown in Supplementary Video S1. However, to note, the arrangement is not defect free, which we think, is not required for present application. When it was ready, another glass coverslip was coated with thin film of Poly(dimethyl)siloxane (PDMS) (Dow Corning, Sylgard 184) by spinning (1:10:100:: prepolymer:curing agent:n-Hexane (v/v)) on it at 1000 rpm for two min using Spin NXG-P2. This plate of self-assembled beads was heated at 60 °C for 12 h. This process transferred the beads on the PDMS and made the arrangement permanent. Then the beads stamp was coated with diluted PDMS for two min at 1000 rpm. The PDMS coating was cured by placing the stamp in Hot air oven (60 °C) for 12 h. The stamp was then sonicated in 70% ethanol followed by Milli-Q water and used for microcontact printing.

For PDMS cylinder stamps (Fig. 5B), syringe needles (BD precision needles) of 26, 24, 22 and 18 gauges were used. 10:1 ratio of prepolymer: curing agent by weight was thoroughly mixed and degassed in vacuum desiccator till there were no air bubbles. Then by using a syringe, the PDMS solution was injected into the needle carefully avoiding any air bubble to form inside. Then the needles filled with PDMS was kept in a Hot air oven at 60 °C overnight for curing. The cured PDMS was pulled out of the needles by holding the PDMS drop formed at the tip of the needles. 8–9 PDMS cylinders of the length 1 cm were arranged parallel and attached on the dilute PDMS coated coverslip, prepared as described before. The stamp was incubated in hot air oven for 12 h at 60 °C and cleaned in sonicator as mentioned earlier.

The stamps prepared using the above methods is shown in Fig. 5C. The 60 µm diameter stamp (Fig. 5C-I) fabricated using conventional soft lithography was used as a control for further experiments. The polystyrene bead stamp (Fig. 5C-II) and 975 µm and 375 µm diameter PDMS cylinder stamps (Fig. 5C-III and IV, respectively) along with the control were used for patterning protein on various substrates.

### Microcontact printing (µCP) of protein on substrate

**Figure 6:**
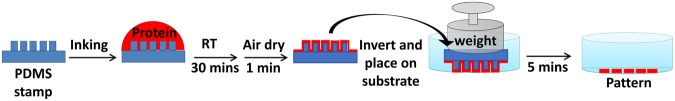
Schematic of Microcontact printing (µCP).

The stamp prepared as described in the previous section were used for µCP as illustrated Fig. 6. The stamps were incubated with 50 µg.ml^−1^ FITC-conjugated collagen I (col-I) (Sigma Aldrich, Cat. no. C4361) for 30 min at room temperature. The excess protein solution was removed from the stamp, and the stamp was air dried for 1 min. The stamp was then placed onto the substrates, non-tissue culture plastic dish (Tarsons, Cat. no. 460035) or PDMS of varying crosslinking density, patterned side down and appropriate weight (20 g, 50 g, 100 g) was applied on top of the stamp. This ensured conformal contact between stamp and substrate to allow complete transfer of the protein. The substrate was then immediately submerged in PBS (pH = 7.2) to avoid drying of protein and removal of excess protein. These substrates can be stored at 4 °C until further use for cell culture but not more than 1 week. Ratios of Sylgard prepolymer to the cross-linking reagent as 10:1, 15:1 and 20:1. Modulus of the substrate thus prepared were estimated using rheometer (Supplementary Fig. S5) as 2.5 MPa, 1.5 MPa, and 1 MPa, respectively. In this article, these substrates are referred by their stiffness.

### Cell line and cell culture

Experiments were performed using mouse myoblast cell line, C2C12, generously donated by Dr. Jyotsna Dhawan, InStem. The cell line was cultured in Dulbecco’s Modified Eagle Medium (DMEM) (Invitrogen), and 10% Fetal Bovine Serum (FBS) (Gibco) supplemented with 1X Antibacterial-Antimycotic (Invitrogen), 1X Glutamax (Invitrogen) in a humidified CO_2_ incubator at 37 °C. Cells were passaged at 70–80% confluency using 0.5% Trypsin-EDTA (Invitrogen) for 3 min at 37 °C to detach the cells and centrifuged at 1000 rpm for 5 min. Cell count was taken using Evos Countess and seeded on the substrates or for maintenance.

### Cell seeding on substrates

Before cell culture, the substrates were passivated with 1% Pluronic F-127 (Sigma, Cat. No. P2443) in PBS for 10 min at room temperature to prevent non-specific cell adhesion. 5000 cells.cm^−2^ were seeded on the printed substrates in serum-free medium for 30 min and incubated at 37 °C. After the cell attachment and initial spreading, the floating cells were removed, and fresh complete media was added and kept in a CO_2_ incubator at 37 °C.

Microscopy was performed after 24 h, 48 h to check the cell patterning using Evos Fl Auto (Life Technologies) fluorescence microscope.

### Cell viability assay

Cells pattern on the rectangular print was used for viability check after 24 h of cell seeding. Cells were incubated with complete media containing Calcein AM (1:2000) (Life Tech, Cat No. C3099) and propidium iodide (PI) dye (1:100) for 10 min at 37 °C. Nucleus was stained using Hoechst (1:10000) (Life Tech. Cat No. H3570). After incubation, the media was replaced with Opti-MEM (Invitrogen) and images were captured using EVOS FL Auto.

### Image analysis and Statistics

Images of FITC-conjugated collagen protein pattern were analyzed using Image J software. All the data come from more than 3 experiments and more than 6 cylinders. At least 3 widths were measured along the length of one cylinder.

## Electronic supplementary material

Supporting Information

Supplementary Video

## Data Availability

All data generated or analyzed during this study are included in this published article (and its Supplementary Information Files).
