# Supplementary material for: Low Cost and Lithography-free Stamp fabrication for Microcontact Printing
Source: Sci Rep. 2019 Jan 31;9:1024. doi: 10.1038/s41598-018-36521-x (PMC6355877; doi:10.1038/s41598-018-36521-x)
Supplement: Supplementary file 1 — Supporting Information [file 41598_2018_36521_MOESM1_ESM.docx]

Supplementary Information

Low Cost and Lithography-free Stamp fabrication for Microcontact Printing

Akshada J. Khadpekar^#1^, Moin Khan^#1^, Abhishek Sose^1^, Abhijit Majumder*^1^

^1^ Indian Institute of Technology Bombay, Mumbai-400076, India

^#^ Equal Contribution

*Author for Correspondence: [abhijitm@iitb.ac.in](mailto:abhijitm@iitb.ac.in)


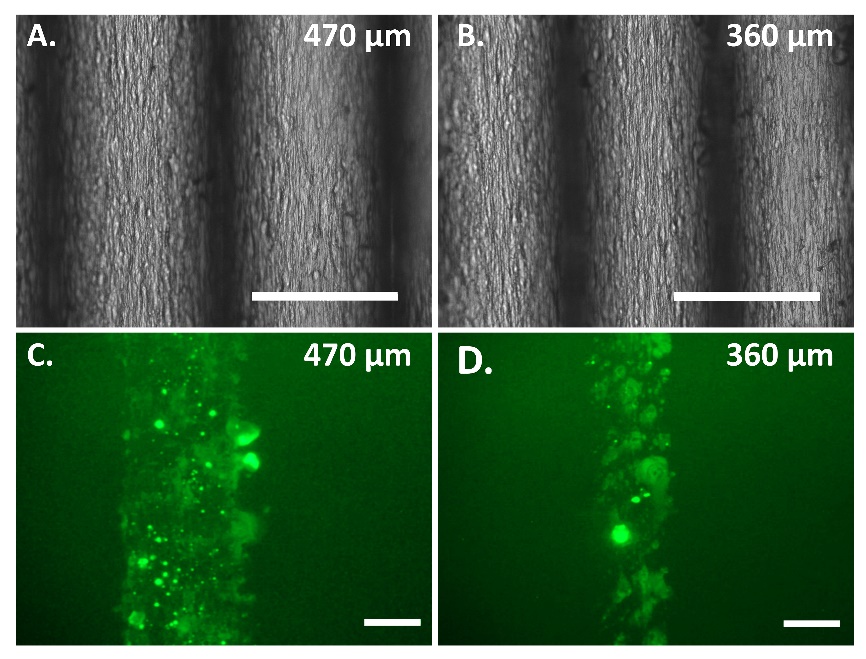


**Supplementary Figure S1**. Printing is feasible with other gauge needles. PDMS cylinders fabricated using 22 and 26 gauges were used for µCP. The diameter of the PDMS cylinders of the 22 and 26 gauges was 470 µm and 360 µm, respectively. Rectangular pattern of FITC-conjugated collagen-1 was obtained on plastic by applying 100 g weight on 500 µm (A) and 300 µm (B) stamps. Scale bar for A-B= 400 μm and for C-D= 100 μm


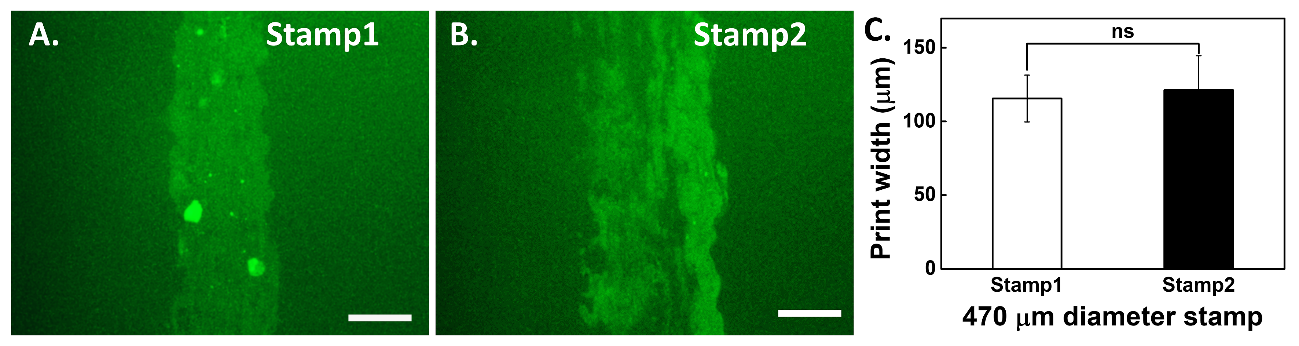


**Supplementary Figure S2**. Comparison of pattern obtained from same size stamps with different fabrication cycle: Two different stamps, stamp1 (A) and stamp2 (B), fabricated using same size needles (22 gauge) were used to print protein on plastic dish with constant load (50 g) applied on the stamps. There was no significant difference between the print width obtained from two individually fabricated same size stamps. Scale bar = 100 μm


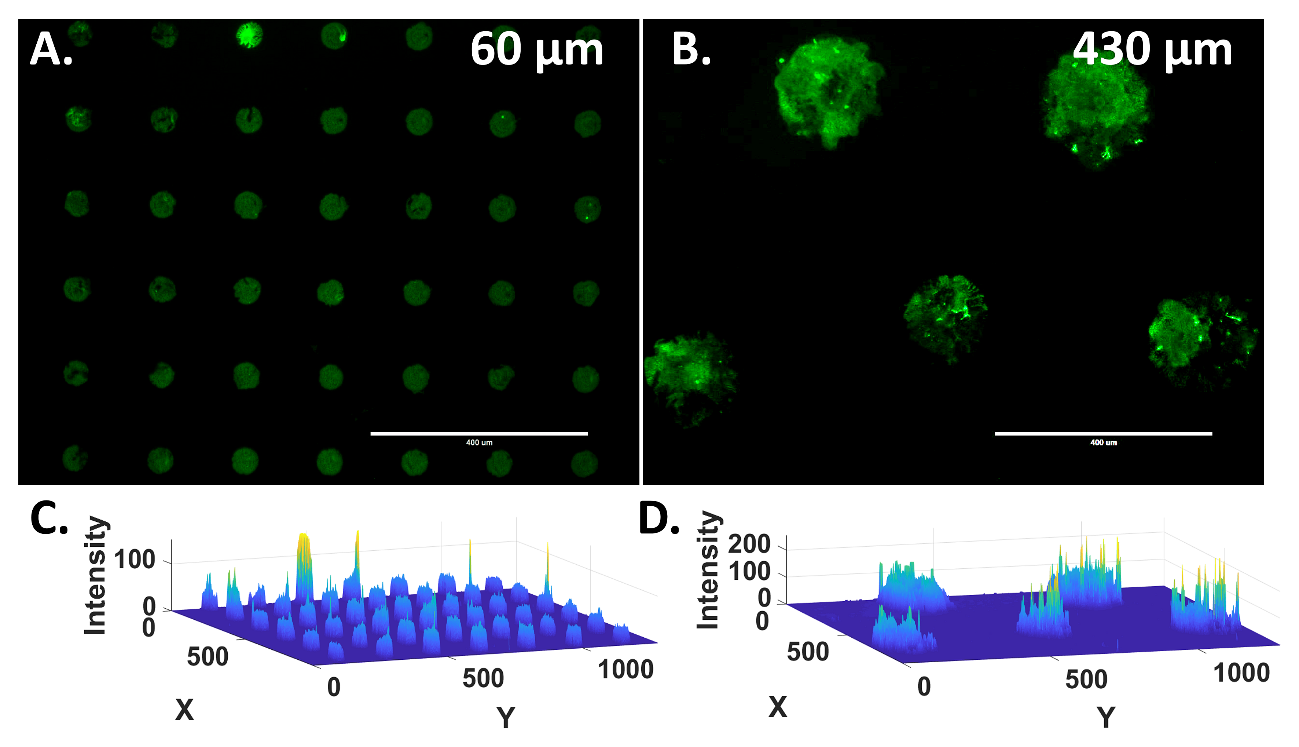


**Supplementary Figure S3.** Comparison between mould and control stamp: Protein islands obtained using 60 μm diameter lithography fabricated (A) and 430 μm diameter beads stamp (B) on 1 MPa PDMS substrate using 20 g applied weight. The surface intensity plot for the corresponding 60 μm (C) and 430 μm diameter protein islands.


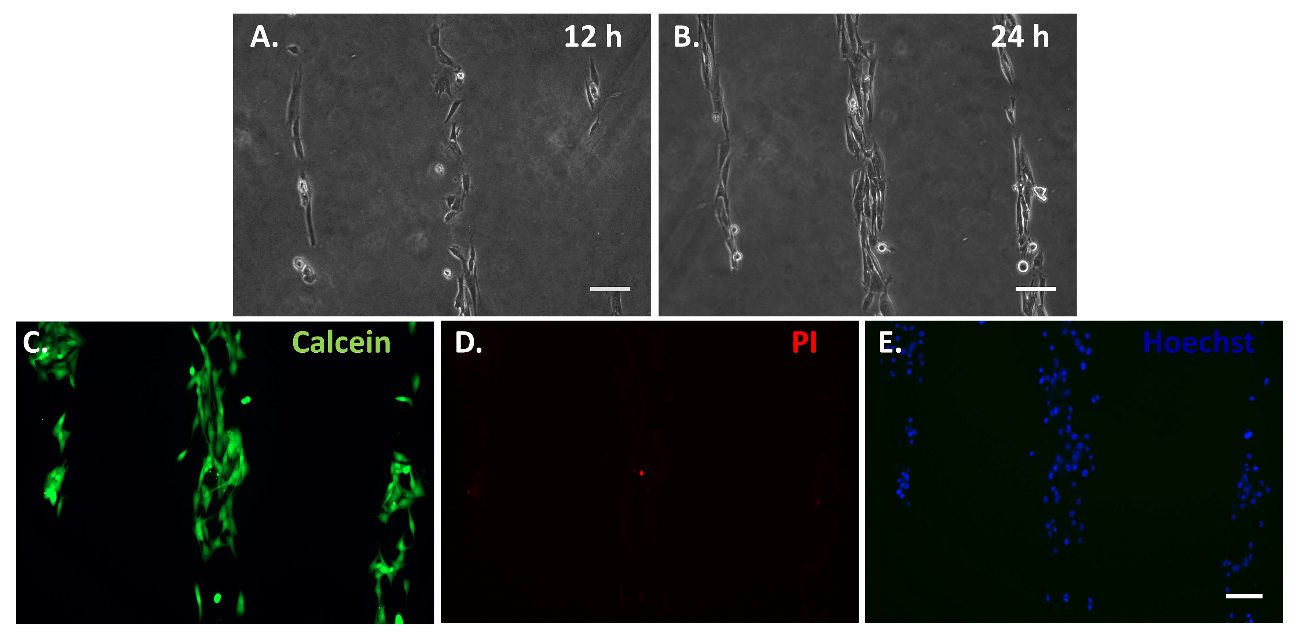


**Supplementary Figure S4**. Viability of cells on patterns: Phase contrast images of C2C12 cells on print obtained using 375 μm stamp with 20 g weight on plastic after 12 h and 24 h of cell seeding. Further, we performed calcein/PI staining. All the cells on the pattern are viable as they are all calcein positive (C) and PI negative cells (D). We also stained the nucleus of the cells using Hoechst dye (E). Scale bar = 100 μm


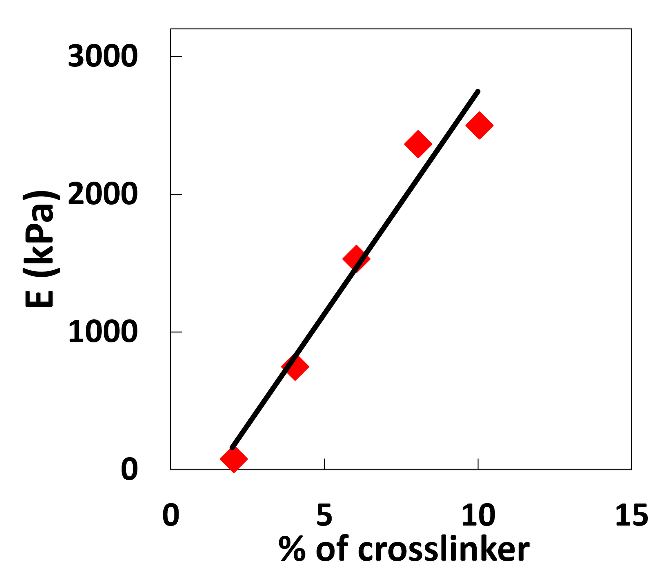


**Supplementary Figure S5.** A standard curve of Young’s modulus (E) of poly-(dimethylsiloxane) (PDMS): Young’s modulus of PDMS substrates with 2%, 4%, 6%, 8% and 10% cross-linking reagent by weight was measured using rheometer by setting amplitude to 0.05% and 0.1 to 10 Hz frequency.

**Supplementary Video S1.** The process of hexagonal packing of 430 μm polystyrene beads.
